# Protective Effect of Quercetin on Chloroquine-Induced Oxidative Stress and Hepatotoxicity in Mice

**DOI:** 10.1155/2013/141734

**Published:** 2013-03-27

**Authors:** Shrawan Kumar Mishra, Prabhat Singh, Srikanta Kumar Rath

**Affiliations:** Genotoxicity Laboratory, Toxicology Division, CSIR-Central Drug Research Institute (CDRI), Lucknow 226 001, India

## Abstract

The present study was aimed to find out the protective effect of quercetin on hepatotoxicity resulting by commonly used antimalarial drug chloroquine (CQ). Swiss albino mice were administered with different amounts of CQ ranging from human therapeutic equivalent of 360 mg/kg body wt. to as high as 2000 mg/kg body wt. We observed statistically significant generation of reactive oxygen species, liver toxicity, and oxidative stress. Our observation of alterations in biochemical parameters was strongly supported by real-time PCR measurement of mRNA expression of key biochemical enzymes involved in hepatic toxicity and oxidative stress. However, the observed hepatotoxicity and accompanying oxidative stress following CQ administration show dose specific pattern with little or apparently no effect at therapeutic dose while having severe effects at higher dosages. We further tested quercetin, an antioxidant flavanoid, against CQ-induced hepatoxicity and found encouraging results as quercetin was able to drastically reduce the oxidative stress and hepatotoxicity resulting at higher dosages of CQ administration. In conclusion, our study strongly suggests co administration of antioxidant flavonoid like quercetin along with CQ for antimalarial therapy. This is particularly important when CQ is administered as long-term prophylactic treatment for malaria as chronic exposure has shown to be resulting in higher dose level of drug in the body.

## 1. Introduction

Chloroquine [7-chloro-4-(4-diethylamino-1-methylbutylamino) quinoline, CQ] is a 4-aminoquinoline derivative antimalarial compound. Apart from being an established antimalarial, CQ also finds use as an anti-inflammatory drug in the treatment of rheumatoid arthritis [1–3], discoid lupus erythematosus [4, 5], and amoebic hepatitis [6]. In spite of reports of chloroquine resistance in many parts of the world, it is still used as first line of therapy against malaria in many developing countries owing to its being readily available and cheaper. 

However, despite being effective in range of diseases, its use was always under scrutiny as it has narrow safety margin and has shown wide range of side effects including cardiac and neurological disorders, retinopathy, and ototoxicity [7–9]. Severe liver injury and hepatitis in the presence or absence of systemic features have also been described in CQ users [10–12]. Liver being the largest gland and major site for drug metabolism has aroused considerable interest among researchers, and some studies were conducted in the past that have addressed the chloroquine-induced hepatotoxicity [13, 14]. However, several studies [13–15] advocate that hepatotoxicity caused by CQ is mainly due to its oxidative potential. However, there have been few other reports [16, 17] that consider malaria infection as such a cause for oxidative stress and propose antioxidant action of CQ for its antimalarial property. Furthermore, in contrast to previous two distinct convictions, there are reports [18, 19] that say that CQ has little or no role in oxidative stress in malaria-infected or healthy animals. Quercetin (3,5,7,3′,4′-pentahydroxyflavone), one of the most widely distributed flavonoids in plants, is abundant in onions, kale, broccoli, apples, berries, Ginkgo Biloba, tea, and red wine. There have been several studies which have established quercetin possessing an excellent efficacy of scavenging free radicals [20], which is stronger than the traditional antioxidants Vitamin C and Vitamin E [21–23]. Quercetin has been applied for clinical therapy because of its multiple pharmacological activities, such as suppression of cell proliferation, protection of LDL oxidation, prevention of platelet aggregation, and induction of apoptosis [24]. It has been hypothesized that these protective effects of quercetin might be due to a wide variety of mechanisms, including reducing oxidative stress by scavenging free radicals, promoting cellular survival by modulating intracellular signals, or decreasing toxicities of xenobiotics and carcinogens by regulating gene expression [25].

Quantitative real-time PCR is a powerful technique that has been utilized extensively for detection of finer gene expression changes at transcript level. Present study utilizes high efficiency lightCycler 480 (Roche Diagnostics) real-time PCR technology along with conventional biochemical enzymatic analysis to evaluate the mechanistic details of chloroquine-induced hepatotoxicity. The specific aims of the present study were therefore (1) to investigate the effect of dose-dependent chloroquine administration in liver of Swiss albino laboratory mice; (2) to assess the efficacy of quercetin, an antioxidant flavonoid, in counteracting CQ-mediated hepatotoxicity; (3) to understand the mechanism of CQ-mediated hepatotoxicity and its possible amelioration by quercetin at gene expression and enzymatic activity level.

## 2. Materials and Methods

### 2.1. Chemicals

Kits for estimation of alanine aminotransferase (ALT) and aspartate aminotransferase (AST) are obtained from Beckman Coulter, Ireland. All other chemicals and biochemicals were of analytical grade and unless otherwise stated were obtained from Sigma Chemical Company (St. Louis, MO, USA).

### 2.2. Treatment of Animals

Swiss albino male mice (20–25 g) were procured from laboratory animal division of the institute. The institutional ethics committee for the use of laboratory animals approved the study. The animals were kept in the animal house under standard conditions of temperature and humidity (temperature: 22 ± 2°C; humidity: 45–55%; light intensity: 300–400 lx). The animals were supplied proper pellet diet and water ad libitum. The doses for CQ and quercetin were decided based on the available literature [13, 26, 27], and therapeutic dose in mice (360 mg/Kg) equivalent to that used in human was calculated based on equivalent surface area index [28–32]. Other dosages higher to that of therapeutic equivalent dose were selected as having maximum effect on the level of markers of hepatotoxicity without causing death. Animals were divided into six groups each consisting of six animals and were given following dosages orally:

 Group  1: (control) received 1% DMSO,
 Group  2: (therapeutic equivalent dose) received 360 mg/kg body wt. of CQ
 Group  3: received 1000 mg/kg body wt. of CQ,
 Group  4: received 2000 mg/kg body wt. of CQ,
 Group  5: received 50 mg/kg body wt. of quercetin followed by CQ 2000 mg/Kg body wt.,
 Group  6: received 50 mg/kg body wt. of quercetin.

All animals were sacrificed by cervical dislocation after 24 h of treatment. Liver was taken out after perfusion with normal saline and kept at −70°C deep freezer.

### 2.3. Blood Collection, Serum Biochemistry, and Estimation of Intracellular Reactive Oxygen Species (ROS)

Blood was taken out from each animal through cardiac puncture and allowed to stand undisturbed for 30 min. The serum was separated by centrifugation at 2,000 rpm for 15 min in a Minifuge (Sigma). The serum levels of alanine aminotransferase (ALT) and aspartate aminotransferase (AST) were estimated using an automated biochemical analyzer (Beckman, Coulter, CA, USA). Intracellular ROS in peripheral blood mononuclear cells (PBMCs) was analyzed using the fluorescent probe 2′,7′-dichlorofluorescein-diacetate (H2DCFDA), a nonfluorescent compound under normal condition, which is converted into highly fluorescent dichlorofluorescein (DCF) by cellular peroxides. Blood samples (1 mL) collected from mice were placed in heparinized tubes and stratified on Histopaque (Sigma Diagnostics, USA) followed by centrifugation to collect buffy coat containing PBMC. Subsequently, 10 *μ*L of 1.25 mM H2DCFDA (Sigma, USA) in methanol was added to PBMC for ROS estimation. Samples were incubated for 30 min at 37°C. Cell-associated fluorescence was monitored on fluorescence activated cell sorter (FACS) Caliber flow cytometer (Becton Dickinson, San Jose, CA) with the excitation wavelength at 488 nm and emission wavelength at 530 nm. Data was analyzed by “CellQuest Pro” software (Becton Dickinson, USA), and the differences in the mean fluorescence intensity (MFI) were calculated.

### 2.4. Histopathological Examinations of Liver Sections

Fixed liver tissues were washed overnight, dehydrated through graded alcohols, and embedded in paraffin wax. Serial sections of 5 *μ*m thickness were stained with haematoxylin and eosin (H&E) for histological examination as per our previous studies [29, 33].

### 2.5. Biochemical Examination of Liver Tissue

The frozen liver tissue samples were quickly weighed before being homogenized in ice-cold 50 mM potassium phosphate buffer (pH = 7.4) containing 1 mM EDTA. The homogenates were then centrifuged at 10,000 ×g for 30 min at 4°C. The supernatants were separated and used for enzyme activity assays.

### 2.6. Measurement of Lipid Peroxidation Level (LPO)

Lipid peroxidation was estimated by using the method of Ohkawa et al. [34]. A 10% tissue homogenate in SDS (10% w/v) was incubated for 5 min; glacial acetic acid (20%) was added and the mixture was further incubated for 2 min. TBA (0.8%) was added to the reaction mixture, which was incubated in a boiling water bath for 1 h. After centrifugation at 10,000 ×g for 5 min at 4°C, the supernatant was collected; absorbance was recorded at 532 nm against the control blank.

### 2.7. Catalase (CAT) Activity Measurement

Catalase was measured by the method as described by Sinha [35]. In brief, assay mixture consisting of 0.01 M phosphate buffer (pH 7.0), 0.2 M hydrogen peroxide, and tissue homogenate was incubated at 37°C for 1 min. The reaction was stopped by the addition of potassium dichromate (5% w/v) and acetic acid. The remaining hydrogen peroxide was determined by measuring chromium acetate after heating the assay mixtures in a boiling water bath for 15 min. The absorbance was read at 570 nm against control without hydrogen peroxide. The enzymatic activity was measured in *μ*mol/min/mg protein.

### 2.8. Superoxide Dismutase Activity (SOD) Measurement

Superoxide dismutase (SOD) was measured spectrophotometrically at 570 nm using modified method of Kakkar et al. [36]. In brief, assay mixture containing sodium pyrophosphate buffer (pH 8.3, 0.052 M), phenazine methosulfate (186 *μ*M), nitroblue tetrazolium (300 *μ*M), NADH (780 *μ*M), and appropriately diluted enzyme in total volume of 3 mL was incubated at 37°C for 90 s. The reaction was stopped by the addition of glacial acetic acid. The reaction mixture was mixed vigorously by adding n-butanol and was allowed to stand for 10 min before the collection of butanol layer. The intensity of the chromogen in butanol was measured at 560 nm. The SOD activity was calculated in units/mL/min.

### 2.9. Glutathione Reductase (GR) Activity Measurement

Glutathione reductase activity was determined in the tissue homogenate by measuring the decrease in absorbance at 340 nm for 3 min at 30 s intervals [37]. NADPH (2 mM), GSSG (20 mM), and 20 *μ*g cytosolic proteins in 0.2 M phosphate buffer (pH 7.0) were mixed vigorously before absorbance was measured. Enzyme activity is expressed in nmol/min/mg protein.

### 2.10. Glutathione Peroxidase Activity (GPX) Measurement

Glutathione peroxidase activity was determined according to the method of Wendel [38]. The reaction mixture containing 48 mM sodium phosphate, 0.38 mM EDTA, 0.12 mM NADPH, 0.95 mM sodium azide, 3.2 units glutathione reductase, 1 mM glutathione, 0.02 mM DTT, and 0.0007% (w/w) H_2_O_2_ was used to monitor the enzyme activity. Enzyme activity was determined by measuring the decrease in absorbance at 340 nm for 3 min at 30 s interval and expressed in units/mg protein.

### 2.11. Total Glutathione (GSH) Estimation

GSH content was estimated by the method of Ellman [39]. In brief, each reaction consists of 0.6 mM DTNB [(5,5′-dithiobis(2-nitrobenzoic acid)] in 0.2 M sodium phosphate, pH = 8.0, supernatant fraction, and 0.2 M phosphate buffer. The absorbance was measured at 412 nm, and activity was calculated based on a calibration curve plotted using GSH standard.

### 2.12. Protein Measurement

Protein was assayed by the method of Lowry et al. [40], with serum bovine albumin as standard.

### 2.13. RNA Isolation and Real-Time Quantitative PCR Analysis 

Frozen liver tissues from different treatment groups of mice were used for the isolation of RNA using TRIZOL reagent. mRNA was reverse transcribed according to the manufacture's instruction (First Strand cDNA Synthesis Kit for RT-PCR, Invitrogen, CA, USA). PCRs were performed on a LightCycler 480 System (Roche Diagnostics) in 96-well plates. Each reaction was carried out in 20 *μ*L reaction volume comprising SYBR Green qPCR Master Mix (Invitrogen, CA, USA), cDNA template, 200 nM of forward and reverse primers, and nuclease-free water. Serial dilutions of genomic DNA (250–0.08 ng) were used to generate a quantitative PCR standard curve. The LightCycler protocol was 2 min of UDG incubation(Invitrogen, CA, USA) at 50°C followed by 10 min of 95°C hot-start enzyme activation, 40 cycles of 95°C denaturation for 15 s, 60°C annealing and elongation. Melting curve analysis temperatures were 95°C for 5 s, 70°C for 60 s, and then heating to 95°C. Water was used as the template for negative control amplifications included with each PCR run. All reactions were performed in duplicate in each 96-well plate. Data were analysed using the Roche LightCycler 480 software, and Cp was calculated by the Second Derivate Maximum Method. The amount of the target mRNA was examined and normalized to the GAPDH gene mRNA. The relative expression ratio of a target gene was calculated as described by Pfaffl [41], based on real-time PCR efficiencies. Primer pairs are listed in Table 1 and were designed with Lasergene software. Results reported were obtained from at least three biological replicates and PCR runs were repeated at least twice.

### 2.14. Statistical Analysis

Existing data were expressed as the mean ± standard error of the means (S.E.M.). Group means were compared by one-way analysis of variance (ANOVA) with Newman-Keuls postanalysis test. The differences in the data obtained were considered statistically significant when the *P* value was less than 0.05. All statistical analysis was done through using Prism version 5 (GraphPad Software Inc., USA).

## 3. Results

### 3.1. Reactive Oxygen Species (ROS) Level and Serum Biomarkers of Hepatotoxicity

**Figure 1:**
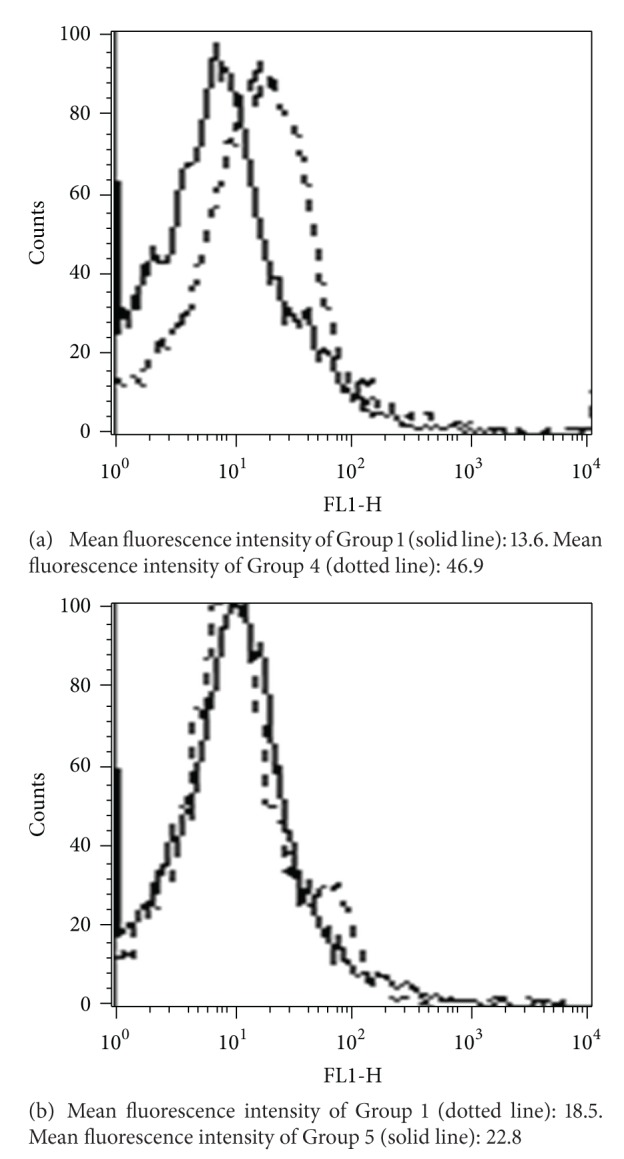
Flow cytometric detection of ROS generation. (a) Group 1 animals that were untreated controls are compared with Group 4 animals that were dosed with 2000 mg/kg body wt. CQ. (b) Group 1 untreated controls were compared with Group 5 animals that were pretreated with quercetin 50 mg/Kg body wt. followed by 2000 mg/Kg body wt. dose of CQ. ROS generation was measured in terms of mean fluorescence intensity.

**Figure 2:**
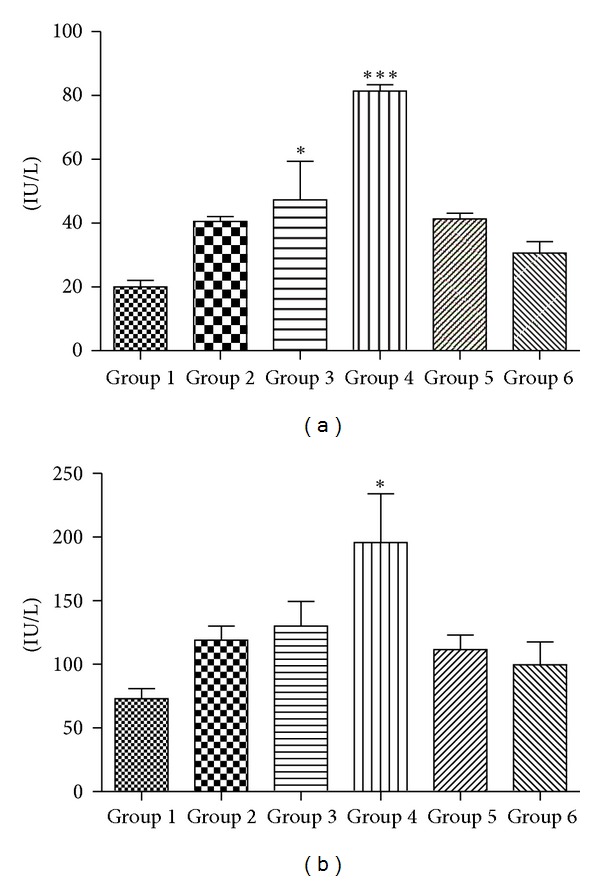
Effect of chloroquine dosing and its protection by quercetin treatment on serum biomarkers of hepatotoxicity (a) alanine aminotransferase (ALT) and (b) aspartate aminotransferase (AST). The data are expressed as means ± S.E.M., and significant changes from untreated control (Group 1) are reported (*(*P* < 0.05), **(*P* < 0.01), ***(*P* < 0.001)).

The potential of chloroquine to generate oxidative stress at different dosage in terms of intracellular ROS production and its consequences following quercetin administration was evaluated. Peripheral blood mononuclear cells isolated from different groups of animals were labeled with H2DCFDA, and increase in mean fluorescence intensity was analyzed by flow cytometry. An approximate 3.45 fold increase of the oxidized DCF mean peak, indicative of higher H_2_O_2_ content, was observed in Group 4 animals treated with 2000 mg/Kg of chloroquine compared with untreated control of Group 1 animals. Quercetin cotreatment (Group 5), however, was effective in about complete reversion of ROS level as mean fluorescence intensity of Group 5 animals was similar to that of untreated Group 1 animals (Figures 1(a) and 1(b)). ROS level was unaltered with respect to Group 1 in all other groups. A significant increase in the activities of serum ALT (*P* < 0.001) and AST (*P* < 0.05) upon chloroquine dosing was observed with activity increasing in direct proportion with CQ dosing. Hepatoprotective nature of quercetin is observed as Group 5 animals showed serum ALT, AST level comparatively closer to that of Group 1 control. Further, treatment of quercetin as such (Group 6) does not seem to influence serum ALT and AST levels (Figures 2(a) and 2(b)).

### 3.2. Lipid Peroxidation

**Figure 3:**
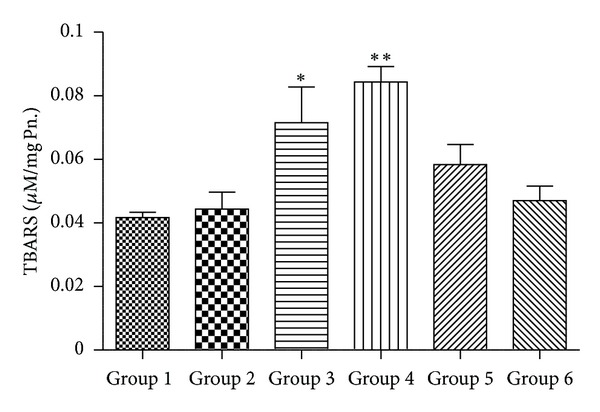
Alterations in lipid peroxidation activity (*n* = 3–5 separate experiments). The data are expressed as means ± S.E.M., and significant changes from untreated control (Group 1) are reported (*(*P* < 0.05), **(*P* < 0.01), ***(*P* < 0.001)).

Figure 3 shows the levels of malonaldehyde (MDA) a secondary product of lipid peroxidation in liver homogenates treated with different dosages of CQ and its consequences following quercetin administration. Group  2 and Group  6 animals do not differ much from untreated Group  1 animals indicating therapeutic equivalent dose of CQ, and quercetin at 50 mg/Kg does not induce lipid peroxidation. However, higher dosages of CQ in Group  3 and Group  4 cause lipid peroxidation as significant (*P* < 0.01) elevation in level of MDA is observed in these cases when compared with Group  1. Quercetin treatment in CQ treated mice (Group  5) reduces significantly the lipid peroxidation incurred due to CQ administration as there was sharp decline in the level of MDA in Group  5 animals when compared with those of Group  4.

### 3.3. Total Glutathione Content

**Figure 4:**
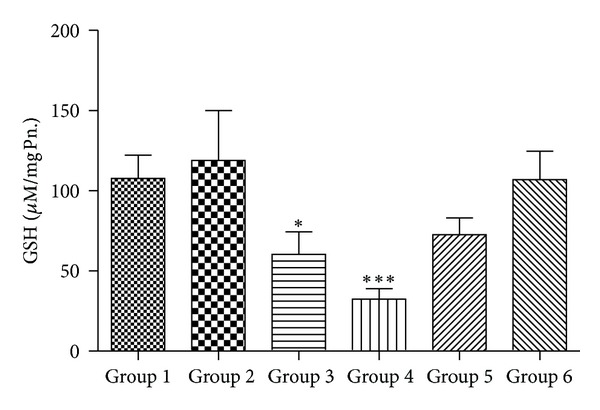
Alterations in reduced glutathione (GSH) content (*n* = 3–5 separate experiments). The data are expressed as means ± S.E.M., and significant changes from untreated control (Group 1) are reported (*(*P* < 0.05), **(*P* < 0.01), ***(*P* < 0.001)).

Figure 4 shows the content of GSH in animals treated with CQ and quercetin. Compared with untreated controls of Group  1 animals, Group  4 animals show statistically significant (*P* < 0.05) reduction in GSH content. Statistically insignificant reduction was also observed in Group  3 animals treated with 1000 mg/Kg body-wt. of CQ. Furthermore, quercetin offers significant protection as GSH content in Group 5 animals was restored to a level close to that of untreated control of Group  1. Quercetin treatment alone (Group  6) did not change the glutathione content.

### 3.4. Activity of Antioxidant Enzymes (SOD, CAT, GPx, and GR)

**Figure 5:**
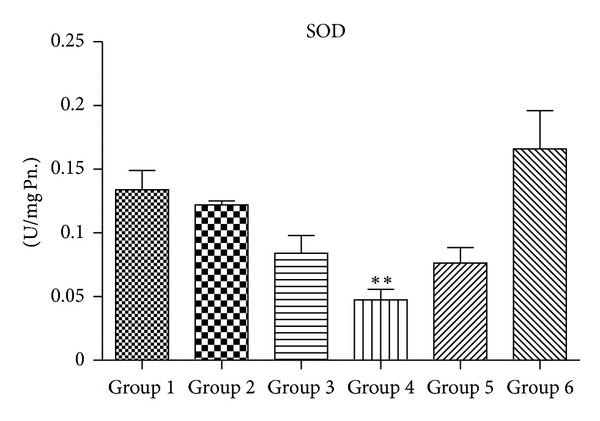
Alteration in superoxide dismutase (SOD) activity (*n* = 3–5 separate experiments). The data are expressed as means ± S.E.M., and significant changes from untreated control (Group 1) are reported (*(*P* < 0.05), **(*P* < 0.01), ***(*P* < 0.001)).

**Figure 6:**
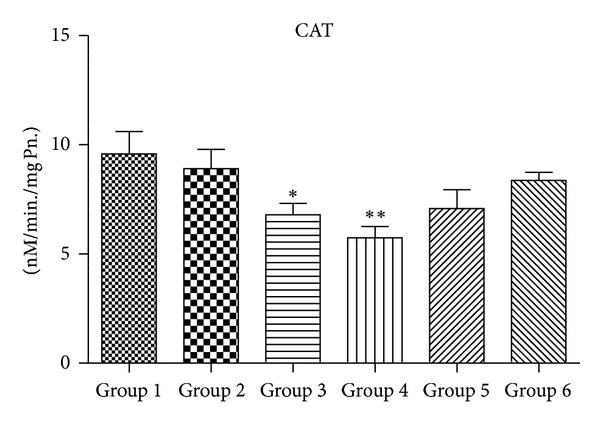
Alteration in catalase (CAT) activity (*n* = 3–5 separate experiments). The data are expressed as means ± S.E.M., and significant changes from untreated control (Group  1) are reported (*(*P* < 0.05), **(*P* < 0.01), ***(*P* < 0.001)).

**Figure 7:**
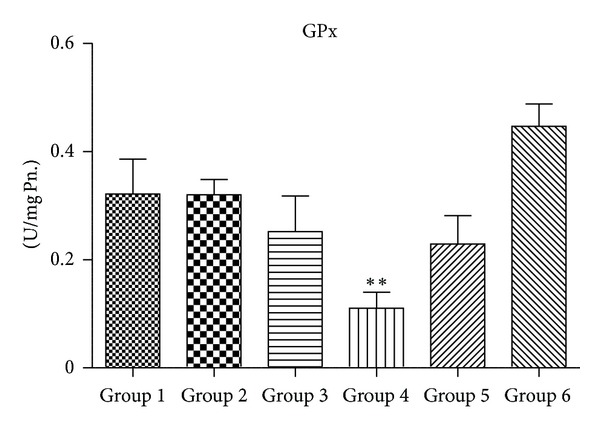
Alteration in glutathione peroxidase (GPx) activity (*n* = 3–5 separate experiments). The data are expressed as means ± S.E.M., and significant changes from untreated control (Group 1) are reported (*(*P* < 0.05), **(*P* < 0.01), ***(*P* < 0.001)).

**Figure 8:**
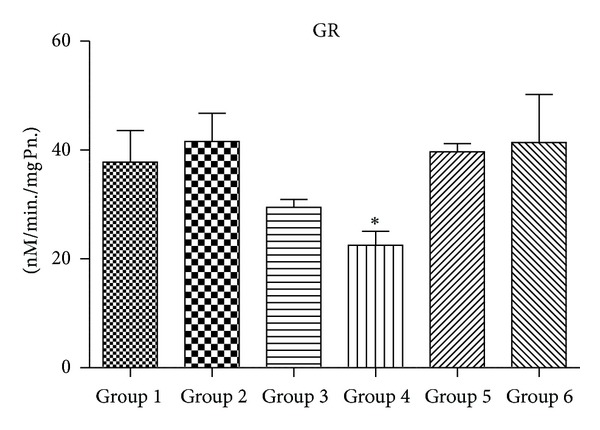
Alteration in glutathione reductase (GR) activity (*n* = 3–5 separate experiments). The data are expressed as means ± S.E.M., and significant changes from untreated control (Group 1) are reported (*(*P* < 0.05), **(*P* < 0.01), ***(*P* < 0.001)).

Figures 5, 6, 7, and 8 demonstrate the activity of SOD, CAT, GPX, and GR, respectively. Group 4 animals treated with 2000 mg/kg body wt. of chloroquine show a statistically significant decrease in the activity of SOD, CAT, GPx, and GR when compared with Group 1 untreated control. Group 3 mice also showed a trend of decrease in all antioxidant enzyme activity, but decrease was only statistically significant in case of CAT activity. Group 2 that received therapeutic equivalent dose of 360 mg/Kg body wt. of CQ and Group  6 that was treated with 50 mg/Kg body wt. of quercetin did not show much different antioxidant profile when compared with Group  1 control animals. Quercetin pretreatment in 2000 mg/Kg body wt. of CQ administered mice (Group  5) shows improvement in antioxidant profile as the activity of SOD, CAT, GPx and GR of Group 5 is closely similar to that of untreated Group 1.

### 3.5. Histopathological Analysis

**Figure 9:**
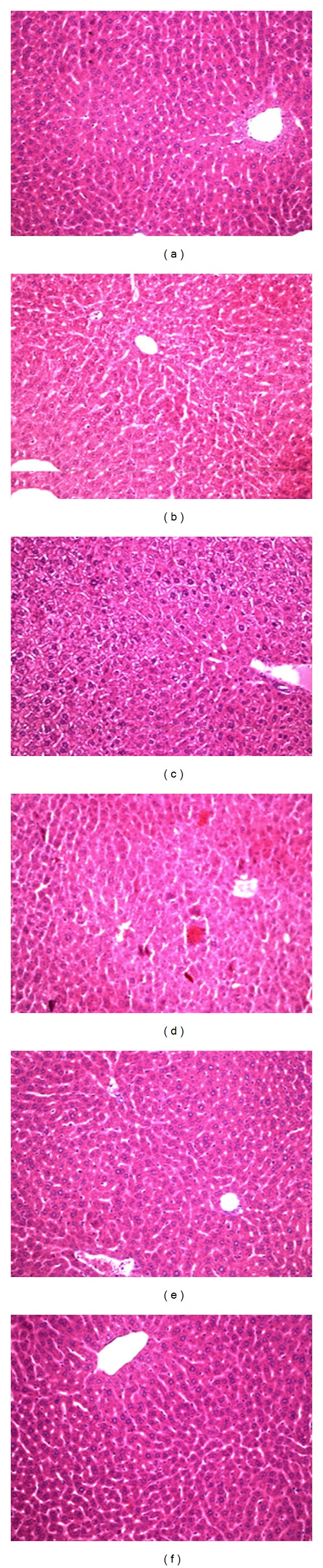
Histopathological evaluation of CQ toxicity in murine liver: (a) Untreated Control (b) treated with 360 mg/kg of CQ (c) 1000 mg/kg CQ treatment (d) treated with 2000 mg/kg of CQ (e) 50 mg/kg quercetin followed with 2000 mg/kg CQ (f) treated with 50 mg/kg of quercetin. Pictures are representative of three different sets of experiments.

Figure 9 shows representative images of haematoxylin and eosin stained mice liver section exposed to chloroquine. Group 4 mice shows significant liver damage as exhibited by pronounced eosinophilic bodies and lymphocyte infilteration. Quercetin treatment (Group 5) is able to reduce the hepatocytic lesions incurred due to chloroquine treatment at 2000 mg/Kg body wt. No significant changes were observed in other study groups.

### 3.6. Quantitative PCR Results

**Figure 10:**
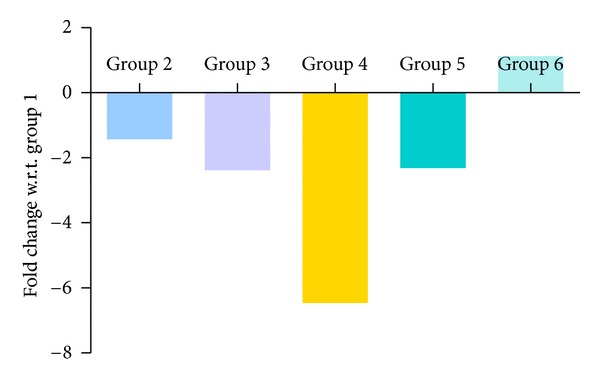
Quantitative PCR measurement of changes in mRNA transcript level of SOD gene. Values were indicative of difference in mRNA fold change of the treated groups with respect to untreated control of Group 1.

**Figure 11:**
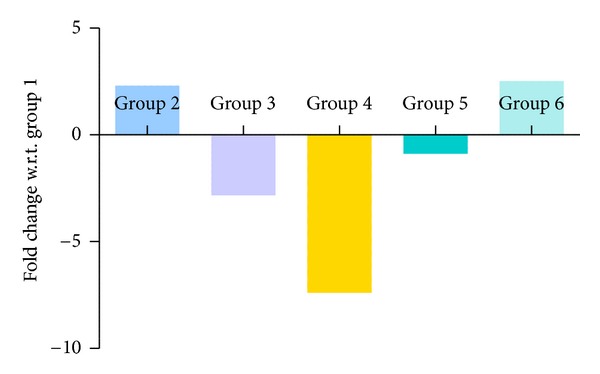
Quantitative PCR measurement of changes in mRNA transcript level of CAT gene. Values were indicative of difference in mRNA fold change of the treated groups with respect to untreated control of Group 1.

**Figure 12:**
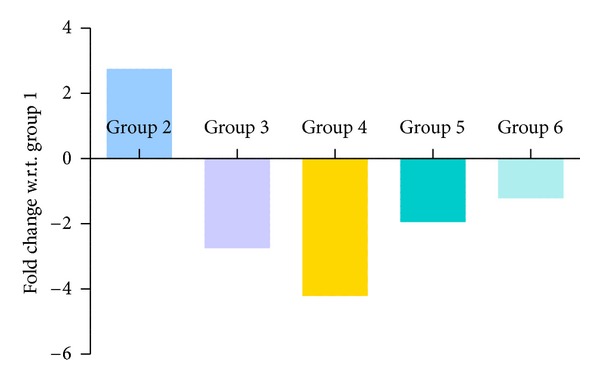
Quantitative PCR measurement of changes in mRNA transcript level of GPx gene. Values were indicative of difference in mRNA fold change of the treated groups with respect to untreated control of Group 1.

**Figure 13:**
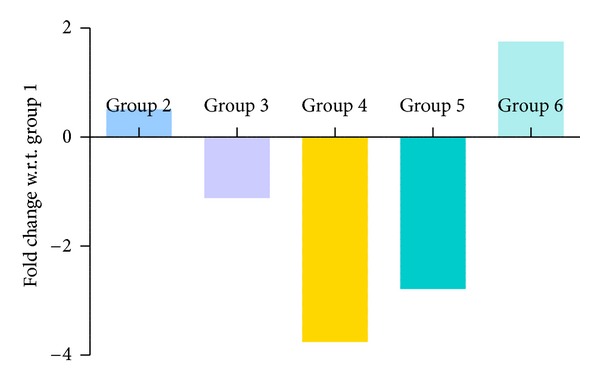
Quantitative PCR measurement of changes in mRNA transcript level of GR gene. Values were indicative of difference in mRNA fold change of the treated groups with respect to untreated control of Group 1.

Figures 10, 11, 12, and 13 show mRNA transcript level of SOD, CAT, GPx and GR genes as measured in terms of Cp value. Group 4 mice show decrease in mRNA level for all the four antioxidant enzymes in the study. Expression profile of Group 2, Group 5 and Group 6 animals was similar to that of untreated control of Group 1. mRNA transcript expression of antioxidant genes is reduced in case of Group 4 when it is compared with Group 1 control. Values in Figures 10, 11, 12, and 13 indicate fold change in mRNA expression with respect to Group 1 control.

## 4. Discussion

Present study evaluates dose specific hepatotoxic effect of CQ and its possible protection by quercetin an herbal flavonoid in Swiss albino mice. Serum alanine aminotransferase (ALT) and serum aspartate aminotransferase (AST) are considered as biomarkers of liver damage [42]; hence, a statistically significant (*P*  value < 0.05) elevation of their levels in Groups 3 and 4 animals indicates CQ-induced hepatotoxicity at these dosages. Group 2 animals treated with therapeutic equivalent dosage did not show any elevation of ALT and AST level when compared to untreated controls of Group 1. The increase in the level of these enzymes in the serum may be due to the injury of tissues with subsequent release of the enzymes into the circulation from the damaged liver tissues [42]. However, our results are in agreement with other studies [13, 14] that had reported similar doses of CQ to be hepatotoxic. Furthermore, increase in blood ROS (reactive oxygen species) level is observed for Group 4 animals when compared with Group 1 controls. This CQ mediated ROS generation may be responsible for oxidative damage in these group of animals. Our observation of amelioration of ROS level by quercetin in Group 5 animals indicates free radical scavenging activity of this plant-based flavonoid.

Since several studies have indicated the development of oxidative stress following liver toxicity [43, 44], we examined the activity and mRNA expression level of major antioxidant systems of the body following chloroquine administration. Lipid peroxidation of unsaturated fatty acids is a commonly used index of increased oxidative stress and subsequent cytotoxicity. It is believed that lipid peroxidation is initiated by the attack of a free radical on fatty acid or fatty acyl side chain of any chemical entity [45] and is regarded as one of the basic mechanisms of tissue damage caused by free radicals. In our study, we observed a significant increase in the concentration of MDA, a secondary product of LPO, in liver of Group 3 and Group 4 mice treated with 1000 and 2000 mg/Kg body wt. of chloroquine, respectively. 

GSH is synthesized in the cytoplasm of the liver cells and then distributed through circulatory/transport system into different organs and subcellular compartments [46]. GSH plays a crucial role in both scavenging ROS and detoxification of drugs and chemicals. GSH reacts directly with ROS and electrophilic metabolites, protecting essential thiol groups from oxidation. Therefore, perturbation in the redox status of GSH cannot only impair cell defense against toxic compounds, but also result in enhanced oxidative stress and tissue injury [46]. We observe a significant decrease in the level of GSH in Group 4 animals when compared to Group 1 control. However, when supplemented with 50 mg/kg body wt. of quercetin, it was able to recoup the loss of GSH content to almost normal. GR and GPx are glutathione-dependent major phase-II drug metabolizing enzymes. We found a decrease in levels of both these enzymes in Group 4 animals in comparison to controls of Group 1. The decrease in GR along with decrease in GSH content suggests a decrease in the overall GSH/GSSG ratio, an index of tissue oxidative stress [47, 48]. 

SOD accelerates the conversion of superoxide radical (O_2_^•^) to H_2_O_2_ [31], while CAT and GPX scavenge H_2_O_2 _and convert it to water [49, 50]. In this study, CQ at the highest dose of Group 4 shows significant decrease in the activity of SOD and CAT when compared with Group 1 animals. Decrease in the activity of SOD in Group 4 animals may be a consequence of decreased de novo synthesis of enzyme proteins characterized by reduction in mRNA transcript level or irreversible inactivation of enzyme proteins from increased free radical production resulting from chloroquine metabolism. Jenkins and Goldfarb have reported that decreased SOD activity reflects oxidative stress [51]. Moreover, decrease in the activity of CAT in Group 4 mice may be due to the excess of superoxide anion radical as a consequence of a reduction in the activity of SOD. Previous reports have also indicated that high production of superoxide anion radical inhibits CAT activity [52]. Again, the decrease in the activity of GPX observed in this study might be the result of decrease in GSH content, a measure substrate in GPX catalyzed reaction. However therapeutic equivalent dose group of Group 2 animals did not show any appreciable ROS generation or decrease in the level of antioxidant enzymes, suggesting that chloroquine at therapeutic dose is quite safe at liver (as serum ALT and AST levels are also not perturbed at this dose) and did not cause oxidative damage.

 Gene expression analysis is a useful tool to study mechanism of drug/chemical-induced toxicity, and it has been extensively utilized in the past to delineate finer details of mechanistic toxicology [53]. A gain or loss of mRNA expression of a particular gene is a profound indicator of accompanying cellular environment of that gene. A real-time qPCR analysis of mRNA expression of major antioxidant enzymes is carried out in present study. Our investigation reveals decrease in mRNA transcript level of major antioxidant enzymes (SOD, CAT, GPX, and GR) in Group 4 animals with respect to that of Group 1 controls. This decrease in expression level might be the effect of generation of ROS and/or development of oxidative stress resulting finally in the decline in enzyme synthesis [54]. 

 In conclusion, we report a loss in activity and reduction in mRNA transcript for major antioxidant enzymes in dose-dependent CQ-treated mice. Further, we also conclude that quercetin, a plant-based flavonoid, has the potential to revert back the CQ-induced toxicity and oxidative stress probably through scavenging the free radical generation. However, we believe that our study is more focused on oxidative stress parameters, and, hence, it is very likely that we may have missed many other relevant pathways that might be important in understanding the finer mechanistic details of CQ-induced hepatotoxicity. A more comprehensive transcriptomic and proteomic profiling through microarray and 2D gel electrophoresis will solve this purpose and, hence, strongly suggested. 

## Figures and Tables

**Table 1:** List of primers used in real-time PCR reaction.

Gene name	Primers
GAPDH	FP-5′-TGGGGTGAGGCCGGTGCTGAGTAT-3′
	RP-5′-CATTGGGGGTAGGAACACGGAAGG-3′

CAT	FP-5′-AAGACCGACCAGGGCATCAAAAA-3′
	RP-5′-AGCGCGGTAGGGACAGTTCACAG-3′

SOD	FP-5′-GGGTTCCACGTCCATCAGTAT-3′
	RP-5′-GCGGCTCCCAGCATTTC-3′

GR	FP-5′-TGCGTGAATGTTGGATGTGTACCC-3′
	RP-5′-CCGGCATTCTCCAGTTCCTCG-3′

GPx	FP-5′-TCACCAACGTGGCCTCGCAATG-3′
	RP-5′-CCTTGATTTCTTGATTACTTCCTGGCTCCTG-3′
